# 

**Published:** 1856-11

**Authors:** 


					﻿Homceopathists in the Massachusetts Medical Society. — A private letter
from Boston, drawn out by our notice of the “Medical World,” in our last
number, assures us that a movement is in progress to rid that venerable
State Society from its present position of fellowship with quackery, in allow-
ing membership to homceopathists. We are glad to learn this fact, and trust
that it may be successful and thorough. That society has too many “nom-
ina digna" in its past and present membership, to soil them by humiliating
associations.
Prof. Timothy Childs, M. D., of Pittsfield, Mass., has been elected to fill
the Chair of Anatomy in the New York Medical College, vice Prof. E. H.
Parker, resigned. Prof. Parker continues in charge of the American Medi-
cal Monthly, which he edits with distinguished ability.
				

